# Autocatalytic Time-Dependent Evolution of Metastable Two-Component Supramolecular Assemblies to Self-Sorted or Coassembled State

**DOI:** 10.1038/s41598-017-02524-3

**Published:** 2017-05-25

**Authors:** Tomoya Fukui, Masayuki Takeuchi, Kazunori Sugiyasu

**Affiliations:** 1 National Institute for Materials Science (NIMS), 1-2-1 Sengen, Tsukuba, Ibaraki 305-0047 Japan; 2 Department of Materials Science and Engineering, Graduate School of Pure and Applied Sciences, University of Tsukuba, 1-1-1 Tennodai, Tsukuba, Ibaraki 305-8577 Japan

## Abstract

Despite substantial effort devoted in the history of supramolecular chemistry, synthetic supramolecular systems still lag behind biomolecular systems in terms of complexity and functionality. This is because biomolecular systems function in a multicomponent molecular network under out-of-equilibrium conditions. Here we report two-component supramolecular assemblies that are metastable and thus show time-dependent evolution. We found that the systems undergo either self-sorting or coassembly in time depending on the combination of components. Interestingly, this outcome, which had been previously achievable only under specific conditions, emerged from the two-component systems as a result of synergistic or reciprocal interplay between the coupled equilibria. We believe that this study sheds light on the similarity between synthetic and biomolecular systems and promotes better understanding of their intricate kinetic behaviors.

## Introduction

A key to enhancing the complexity of synthetic supramolecular systems, ultimately toward the level of biomolecular systems, is to establish a molecular network that consists of many different molecules. In a pool, molecules collide, interact and/or react with each other, and determine whether to self-sort or coassemble as if computing the best outcome^1–15^. It would be intriguing in terms of emergent behavior if the outcome generated from such a mixture of molecules is different from that obtained using individual molecules. In this way, the concept of molecular self-assembly will be expanded to facilitate ever more powerful means of designing new types of smart adaptive materials. To this end, a system under kinetic control offers many opportunities because it may give rise to diverse outcomes depending on the self-assembly pathways rather than the thermodynamic stability^16–39^. Research in this area has attracted increased attention and has recently been documented in several comprehensive reviews^33–35^.

**Figure 1:**
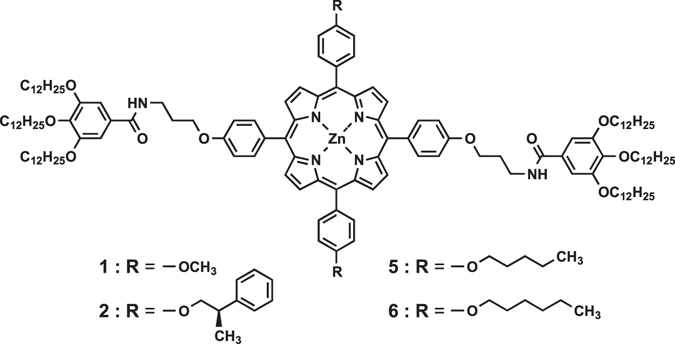
Molecular design. Structures of porphyrin derivatives (**1**, **2**, **5**, and **6**) used in this study.

**Figure 2:**
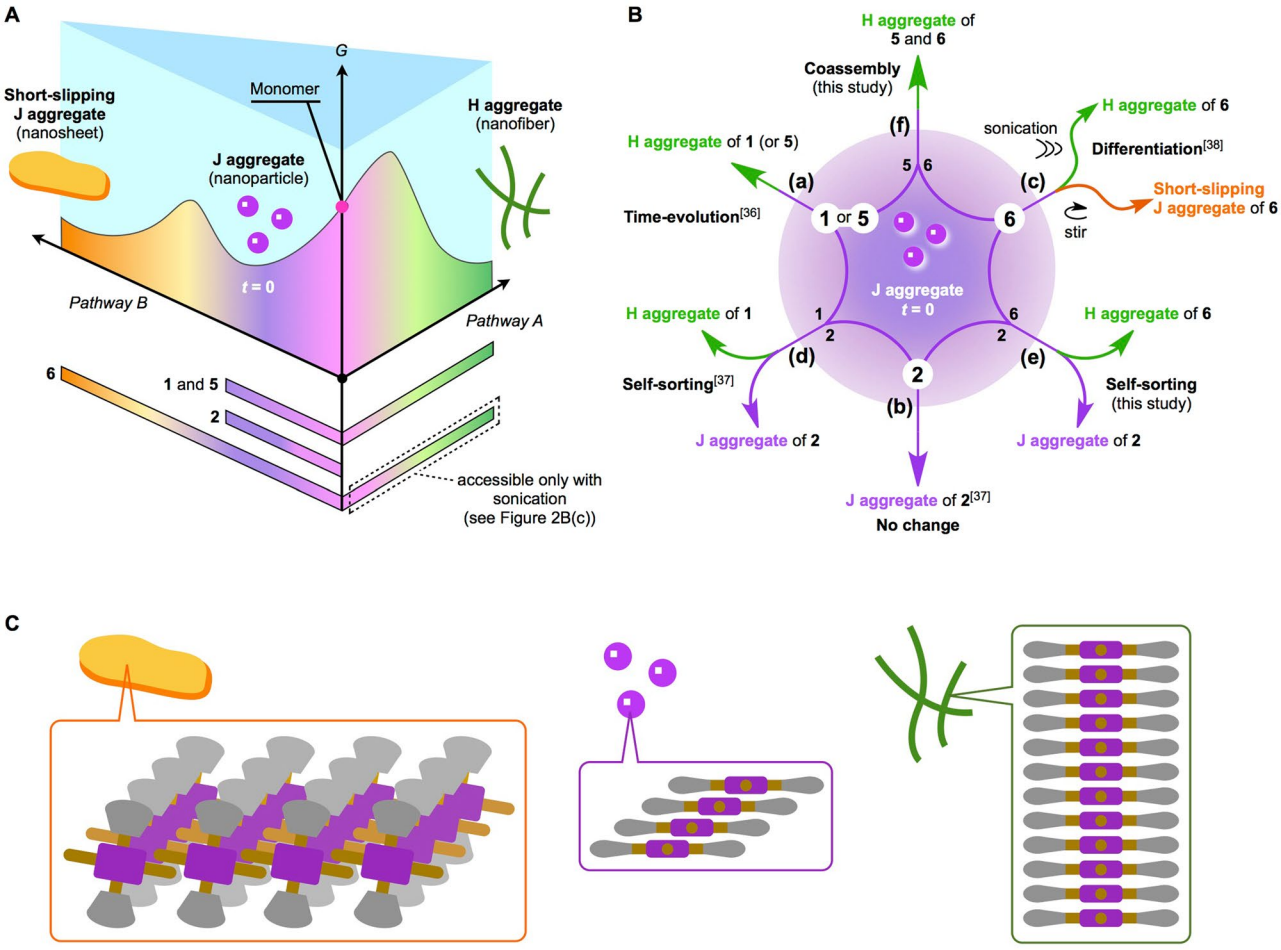
Self-assembly energy landscapes and time-dependent evolution diagram of porphyrin derivatives. (**A**) Energy landscape for self-assembly of porphyrins **1**, **2**, **5**, and **6**; the bars at the bottom indicate the range of pathways allowed for each porphyrin. When a hot solution of porphyrin monomers is cooled, J aggregate nanoparticles are spontaneously formed; that moment is then defined as *t* = 0 in the time-dependent evolution. (**B**) Schematic diagram of time-dependent evolution of metastable J aggregates from center (*t* = 0) outward over time: (a) pure **1** (or **5**)^36, 38^,(b) pure **2** ^37^, (c) pure **6** ^38^, (d) a mixture of **1** and **2** ^37^, (e) a mixture of **2** and **6**, and (f) a mixture of **5** and **6**. The systems in (e) and (f) were investigated in this study. (**C**) Schematic representation of the porphyrin stacking modes in the short-slipping J-aggregate nanosheet, J-aggregate nanoparticle, and H-aggregate nanofibre.

Herein, we report on two-component supramolecular assemblies consisting of porphyrin derivatives (Fig. 1). The porphyrins we used can be categorized into three types according to their self-assembly energy landscapes (Fig. 2A): (a) porphyrins **1** and **5**, (b) porphyrin **2**, and (c) porphyrin **6**. As discussed below, when two of these porphyrins are mixed, a metastable two-component supramolecular assembly is temporarily formed and then shows time-dependent evolution. Interestingly, the systems undergo either self-sorting or coassembly depending on the combination of components (Fig. 2B). Although the systems are still primitive, diversity of outcomes is achieved as a result of kinetic competition and autocatalytic evolution. This study sheds light on the similarity between synthetic and biomolecular systems, as the phenomena are reminiscent of those observed in amyloid fibrillation and other complex processes^40–43^.

When a hot solution of the porphyrin monomer (50 μM in methylcyclohexane (MCH)) was cooled from 373 to 308 K, nanoparticles were spontaneously formed; that moment was then defined as *t* = 0 in the time-dependent evolution. All the kinetic behaviors in this study were investigated under stirring at a rate of 400 rpm unless otherwise noted. Figure 2A shows the energy landscape for self-assembly; the bars at the bottom indicate the range of pathways allowed for each porphyrin (**1** and **5** behave similarly)^36–39^. A nanoparticle of **2** is in the thermodynamically stable state, whereas those of **1**, **5**, and **6** are in metastable states and change into thermodynamically more stable nanostructures (either nanofibers or nanosheets) over time. As demonstrated previously through seeded growth experiments, both nanofibers and nanosheets form in an autocatalytic manner. Absorption spectral measurements indicated that nanoparticle, nanofiber, and nanosheet structures consist of different porphyrin stacking modes, that is, J, H, and short-slipping J aggregates, respectively (Fig. 2C and Supplementary Fig. S1)^44–46^.

When two of these porphyrins were mixed and treated likewise, coassembled J aggregate nanoparticles were formed, which after a lag time showed time-dependent evolution. Figure 2B summarizes the time-dependent evolution of one- and two-component J aggregate nanoparticles as a function of time from the center (*t* = 0) outward, and each molecular system is briefly described below.

(a)^36, 38^ The J aggregate of porphyrin **1** (and that of **5** as well) is in equilibrium with the monomeric porphyrin and is present as a metastable state for a few hours. Once the monomer nucleates the H aggregate, the J aggregate is consumed in elongation of the H aggregate. The transformation shows a sigmoidal kinetics characteristic of an autocatalytic process, and we define the time at which the transformation is 50% complete as *t*_50_ (Supplementary Fig. S2).

(b)^37^ The bulky substituents in **2** prevent face-to-face stacking of porphyrin planes (i.e., H aggregate formation). In addition, **2** does not have a driving force for nanosheet formation (i.e., the short-slipping J aggregate; see below). Therefore, the J aggregate of **2** is incapable of transformation, and thus is thermodynamically stable.

(c)^38, 39^ The metastable J aggregate of **6**, as an on-pathway intermediate, rearranges into the short-slipping J aggregate and assembles into nanosheet structures along pathway B. This process was also found to be autocatalytic and is characterized by the *t*_50_ value (Supplementary Fig. S3). The driving force for nanosheet formation is the van der Waals force among the hexyl chains (this is why porphyrin **2** cannot form nanosheets). However, we found that strong agitation such as sonication facilitated nucleation of the H aggregate, and as a result, the pathway was switched. Now, as in the case of (a), the J aggregate of **6** acts as an off-pathway intermediate and is transformed into the H aggregate along pathway A. Thus, the J aggregate of **6** is capable of differentiation^47^.

(d)^37^ A mixture of **1** and **2** forms a metastable two-component J aggregate, but after a lag time, the system undergoes self-sorting into the H aggregate of **1** and the J aggregate of **2**. An increased proportion of **2** in the mixture impedes H aggregate nucleation of **1**; this is how the *t*_50_ value for the self-sorting process is determined. Using this mechanism, we succeeded in programming the time-dependent evolution. Ghosh *et al*.^14^ and Yagai *et al*.^15^ have recently reported a similar time-dependent self-sorting process.

Having witnessed the unique time-dependent self-sorting of the two-component system (d) and recently discovered the unprecedented differentiation behavior of **6** (c), we sought to investigate the two-component systems in which **6** is involved. We found that:

(e) The metastable two-component **2**/**6** J aggregate undergoes self-sorting into the J aggregate of **2** and the H aggregate of **6**.

(f) The metastable two-component **5**/**6 **J aggregate is transformed into a coassembled **5**/**6 **H aggregate.

These new findings are intriguing considering that the H aggregate nanofiber of **6**, in its pure form, had previously been obtainable only by applying sonication. We assert that pathway complexity plays an important role in determining the final outcome. Although more intricate molecular networks have been reported to date, particularly in the field of systems chemistry^16–20^, the scheme shown in Fig. 2B is rare in that it can be understood in terms of the established energy landscape (Fig. 2A) and hence provides deep insight into molecular self-assembly. In what follows, the results for processes (e) and (f) are described and discussed in detail.

**Figure 3:**
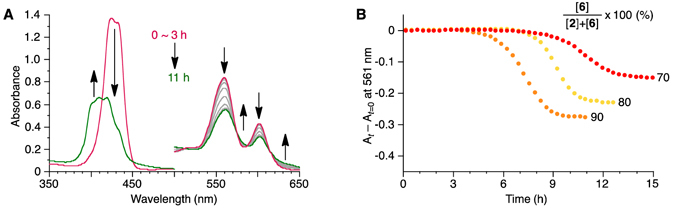
**2**/**6** system. (**A**) Time-dependent absorption spectral changes of two-component **2**/**6** J aggregate: [**2**] + [**6**] = 50 μM; [**6**]/{[**2**] + [**6**]} = 90%. (**B**) Time profile of change in the absorbance at 561 nm, a wavelength that is characteristic of the J aggregate: [**2**] + [**6**] = 50 μM; [**6**]/{[**2**] + [**6**]} = 70, 80, and 90% as indicated.

**Figure 4:**
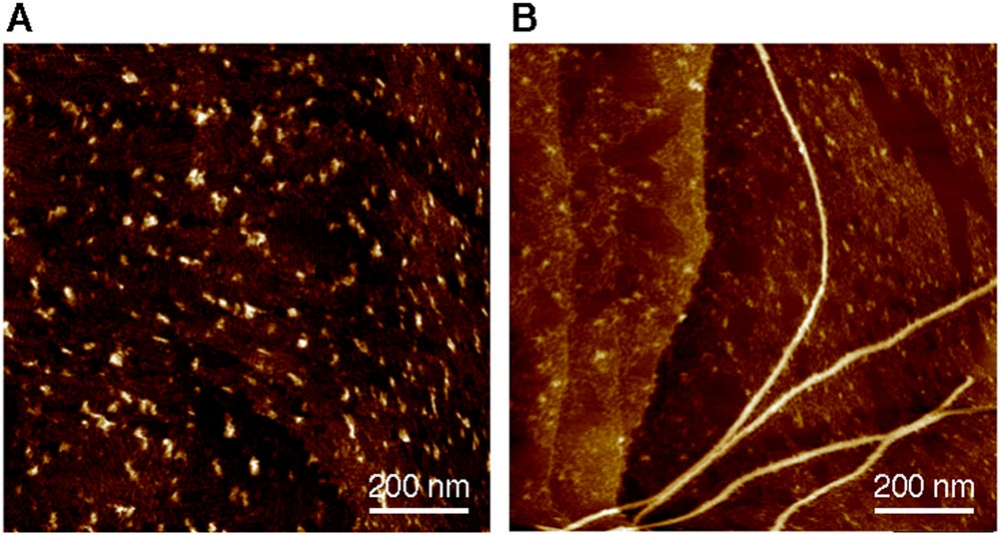
AFM images of two-component **2**/**6** self-assembly. AFM images (HOPG substrate) of (**A**) metastable two-component **2**/**6** J aggregate and (**B**) mixture of H aggregate of **6** and J aggregate of **2** after time-dependent self-sorting: [**2**] + [**6**] = 50 μM; [**6**]/{[**2**] + [**6**]} = 90%.

In our previous study^37^, we confirmed that porphyrins **1** and **2** coassembled into the J aggregate (nanoparticles) as long as the **2** content was less than 30 mol%. Thus, we investigated the time-dependent evolution of **2**/**6** mixtures under such conditions, that is, 10/90, 20/80, and 30/70 molar ratios of the **2**/**6** components. Cooling of a hot MCH solution of the **2**/**6** mixture produced the J aggregate (Supplementary Fig. S4, Supplementary Table S1), and the subsequent time-dependent evolution was probed using absorption spectroscopy. As shown in Fig. 3A, the absorption spectra changed after lag times which were longer than that of the pure **6** J aggregate to transform into short-slipping J aggregates (Supplementary Fig. 3). The plateaued absorbance changes (*A*_*t*_ − *A*_*t*=0_) depended on the concentration of **2**, which suggests that **2** was not involved in the transformation and self-sorted as the J aggregate (Fig. 3B). Absorption spectra revealed that the outcome of the other component, **6**, was the H aggregate despite the fact that it had been accessible only by applying sonication (Supplementary Fig. S5). Atomic force microscopy (AFM) observation also corroborated the formation of the nanofiber structures (Fig. 4).

Why did porphyrin **6** not self-sort as the short-slipping J aggregate? If we were not aware of the “hidden” pathway A that was accessible only using sonication, we might suppose that coexisting **2** induced H aggregate formation of **6** through, for example, a so-called cross-catalytic process. However, Fig. 2A clearly elucidates the mechanism. The short-slipping J aggregate is formed from the on-pathway J aggregate, which suggests that the process is contingent upon the nature of the J aggregate. Remember that the van der Waals force among the hexyl chains in **6** plays a pivotal role in the formation of the short-slipping J aggregate (i.e., nanosheets). Thus, the two-component **2**/**6** J aggregates can be regarded as “mutants” having the sterically demanding moiety in **2** as a defect, and therefore as incapable of forming nanosheets. In the pool, sufficient “pure” J aggregate of **6** cannot statistically be accumulated to form a nanosheet, and instead, coexisting monomeric **6** reciprocally nucleates the H aggregate, thereby biasing the pathway to nanofibers. The fact that the *t*_50_ values became longer with increasing the proportion of **2** also excludes the possibility of the cross-catalytic process. Thus, **6** behaves similarly to **1** (and **5**) in a two-component system with **2**; in fact, the *t*_50_ values of system (e) were comparable with those of (d)^37^.

**Figure 5:**
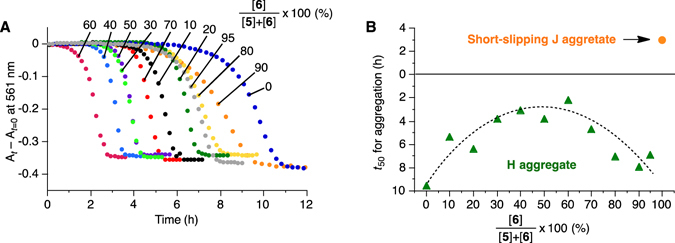
Kinetic studies of the self-assembly behavior of two-component **5**/**6** system. (**A**) Time profile of change in the absorbance at 561 nm, a wavelength that is characteristic of the J aggregate: [**5**] + [**6**] = 50 μM; [**6**]/{[**5**] + [**6**]} = 0, 10, 20, 30, 40, 50, 60, 70, 80, 90, and 95% as indicated. (**B**) Dependence of the mixing ratio of **5**/**6** on the lag time for the transformation of the metastable two-component **5**/**6** J aggregate. Dotted line is just for eye-guide.

**Figure 6:**
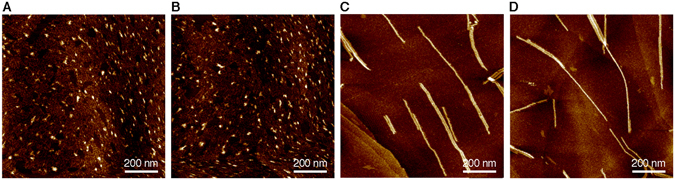
AFM images of two-component **5**/**6** self-assembly. AFM images (HOPG substrate) of (**A**,**B**) metastable two-component **5**/**6** J aggregates and (**C**,**D**) coassembled **5**/**6** H aggregates obtained after time-dependent coassembly: [**5**] + [**6**] = 50 μM; (**A,C**) [**6**]/{[**5**] + [**6**]} = 10%, (**B,D**) [**6**]/{[**5**] + [**6**]} = 90%.

Next, we investigated the two-component system of **5** and **6** (Fig. 2B(f), Supplementary Fig. S6, Supplementary Table S2). Figure 5A shows the time-dependent evolution of the **5**/**6** J aggregates. Again, the final outcome was not the short-slipping J aggregate (nanosheets) but the H aggregate (nanofibers), as evidenced by the absorption spectra (Supplementary Fig. S7) and AFM observation (Fig. 6). Unlike those of the **2**/**6** system, however, the plateaued absorbance changes (*A*_*t*_ − *A*_*t*=0_) of the **5**/**6** system were independent of the mixing ratio, which suggests that all of the J aggregate was consumed (Fig. 5A). Taking the very similar molecular structures into account, it is reasonably assumed that **5** and **6** coassembled into the H aggregate at any mixing ratio. Comparison of the *t*_50_ values as a function of the mixing ratio shows that more “impure” samples exhibit shorter *t*_50_ (Fig. 5B). At present, we do not have a rationale for this tendency, but, considering that the thermodynamic stabilities of the J aggregate were independent of the **5**/**6** ratios (Supplementary Table S2), it should reflect the intricate nucleation mechanisms.

Although the thermodynamic stabilities of the H and short-slipping J aggregates of **6** are comparable^38^, the resultant coassembled **5**/**6** H aggregate was stable for a long time (>a week) without transforming into the short-slipping J aggregate (Supplementary Fig. S8). This is because the metastable J aggregate of **6**, which is the on-pathway intermediate to the short-slipping J aggregate, cannot spontaneously emerge in the “equilibrated” solution. As such, pathway A operates as a “one-way funnel” because of the autocatalytic process. We prepared a pseudo-self-sorted system by mixing solutions of the H aggregate of **5** and the short-slipping J aggregate of **6**. The proportion of the H aggregate gradually increased over time but did not reach unity (Supplementary Fig. S9), reflecting the similar thermodynamic stabilities of H and short-slipping J aggregates. Thus, the coassembled **5**/**6** H aggregate can only be obtained through the metastable **5**/**6** J aggregate under kinetic control.

**Figure 7:**
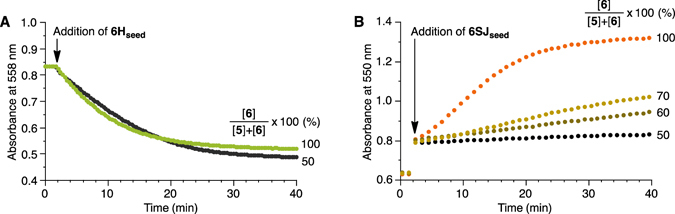
Kinetic studies of seeded growth. Time courses of seeded growth of (**A**) H aggregate and (**B**) short-slipping J aggregate monitored as changes in absorbance at 558 and 550 nm, respectively. Seeds of H and short-slipping J aggregates were prepared from porphyrin **6** as outlined in our previous study and added to the pure **6** J aggregate and two-component **5**/**6** J aggregate: [**6**]/{[**5**] + [**6**]} (%) are indicated in the figures.

To gain further insight into on-pathway versus off-pathway competition, we conducted seeded growth experiments, by which the growth kinetics can be separated from the nucleation kinetics. As outlined in our previous paper^38^, we prepared seeds of the H and short-slipping J aggregates of **6**; these are nanofibers and nanosheets, respectively, but fragmented into small pieces with their porphyrin stacking modes retained. As shown in Fig. 7A, the J aggregates of both **6** and **5**/**6** were converted to the H aggregates upon the addition of an H aggregate seed with similar polymerization kinetics. This result suggests that porphyrins **5** and **6** are indistinguishable along pathway A and are randomly copolymerized. In contrast, the seeded growth kinetics of the short-slipping J aggregate depended on the mixing ratio of **5** to **6** in the J aggregate (Fig. 7B). We infer that the nanosheet growth rate reflects the proportion of the “pure” on-pathway J aggregate in the mixture. It is significant that only 5% of **5** completely prevented spontaneous nanosheet formation (Fig. 5B and Supplementary Fig. S10) while seeded growth of the nanosheet was still observed to some extent even in the presence of 40% of **5**. Thus, the nucleation process appears to play more critical role than the growth process in determining the outcome.

To conclude, it is worth reviewing the diagram shown in Fig. 2B from the viewpoint of pathway competition. For pure **6**, pathways A and B are both allowed, so it appears as though the metastable J aggregate is capable of differentiation. Previously, this differentiation was controlled by mechanical agitation (Fig. 2B(c)), and pathway A was accessible using sonication. In the **5**/**6** system (f), H aggregate nucleation synergistically leads **6** to pathway A, which results in coassembled H aggregate nanofibers. On the other hand, in the **2**/**6** system (e), the presence of **2** prevents pathway B, so **6** is reciprocally guided to pathway A. In both the two-component systems, pathway B was shut down; we therefore assert that the outcome of the on-pathway intermediate is susceptible to changes in the network of molecular self-assembly. As illustrated here, unveiling the entire picture of pathway complexity is important for controlling the outcome of a molecular network. We note that the present system has some similarities with crystal polymorphism and amyloid fibrillation and may promote better understanding and hence control of such intricate phenomena at the molecular level. More practically, the concept of coupled equilibria should be useful to advance programmed supramolecular polymerization and its applications^36, 38, 48–62^.

## Electronic supplementary material

SupplementaryInformation
