# Supplementary material for: Autocatalytic Time-Dependent Evolution of Metastable Two-Component Supramolecular Assemblies to Self-Sorted or Coassembled State
Source: Sci Rep. 2017 May 25;7:2425. doi: 10.1038/s41598-017-02524-3 (PMC5445073; doi:10.1038/s41598-017-02524-3)
Supplement: Supplementary file 1 — SupplementaryInformation [file 41598_2017_2524_MOESM1_ESM.pdf]

Supplementary Information

Autocatalytic Time-Dependent Evolution of Metastable Two-Component Supramolecular Assemblies to Self-Sorted or Coassembled State

Tomoya Fukui[1,2], Masayuki Takeuchi[1,2,*], and Kazunori Sugiyasu[1,*]

[1]*National Institute for Materials Science*
1-2-1 Sengen, Tsukuba, 305-0047 Ibaraki, Japan

[2]*Department of Materials Science and Engineering, Graduate School of Pure and Applied Sciences, University of Tsukuba, 1-1-1 Tennodai, Tsukuba, 305-8577 Ibaraki, Japan*

TAKEUCHI.Masayuki@nims.go.jp
SUGIYASU.Kazunori@nims.go.jp

Table of Contents

1. Materials and Methods page 2
2. Supplementary Figures and Tables page 3
3. Supplementary References page 13

1. Materials and Methods

Unless otherwise noted, reagents and solvents were purchased from commercial suppliers without further purification. Compound **2**[1], **5**[2], and **6**[2] were prepared according to reported procedures. Ultraviolet–visible absorption spectra were recorded using a quartz cuvette of 1.0 or 0.1 cm path length on a Jasco V-630 spectrophotometer equipped with a Jasco ETCS-761 cell holder for temperature control. Atomic force microscopy (AFM) was performed on a Bruker model MultiMode 8 atomic force microscope under ambient conditions in the scan assist analysis. AFM images were analyzed with Bruker Nanoanalysis.

2. Supplementary Figures and Tables

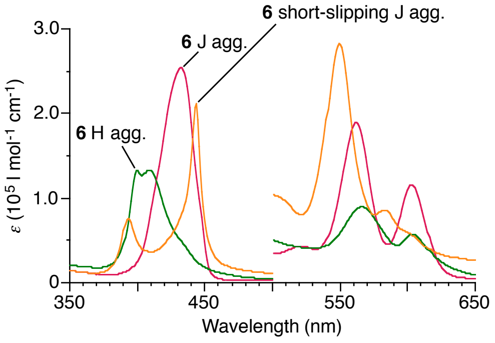

Supplementary Figure S1.
Absorption spectra of J aggregate (pink), H aggregate (green), and short-slipping J aggregate (orange) of **6** in methylcyclohexane[2].

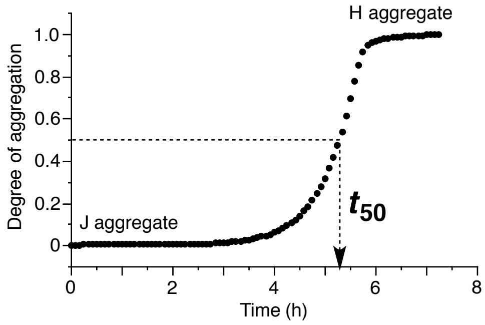

Supplementary Figure S2.
Time profile of changes in the degree of aggregation of **1** H aggregate[1].

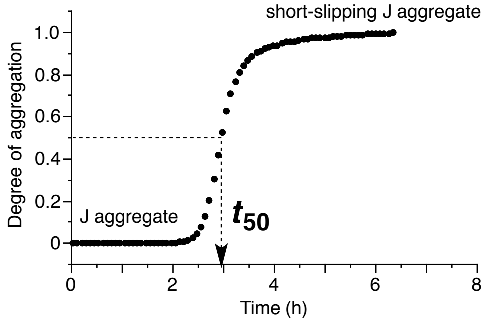

Supplementary Figure S3.
Time profile of changes in the degree of aggregation of **6** short-slipping J aggregate[2].

Thermodynamic analysis of **2**/**6** J aggregate

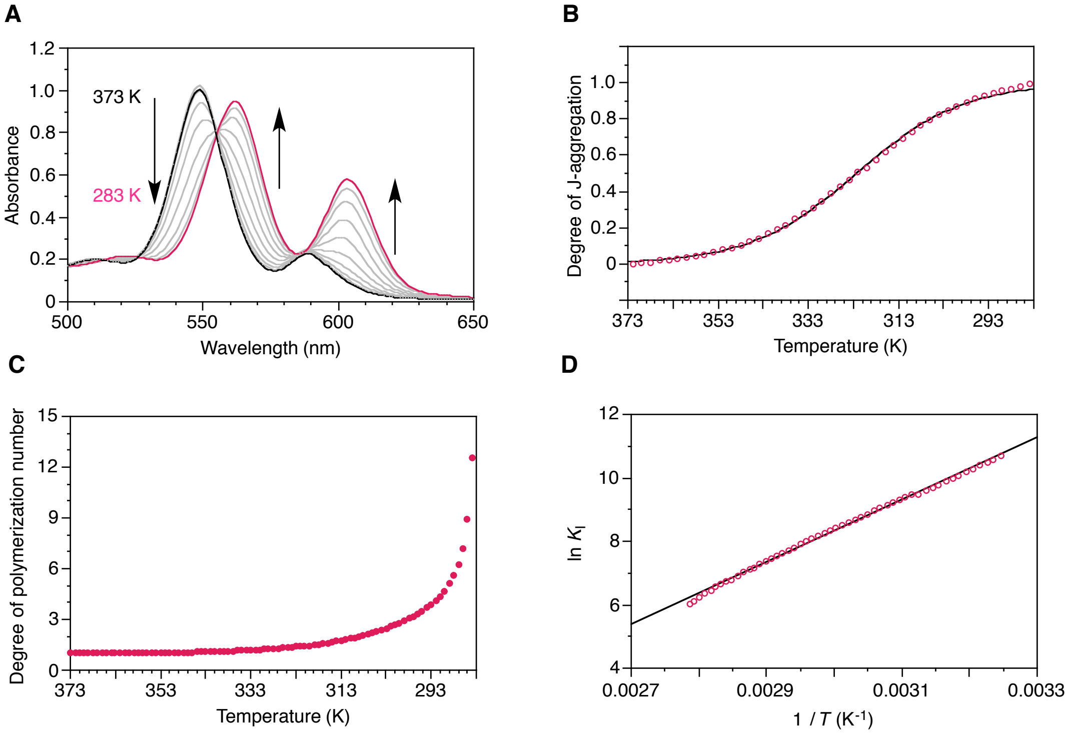

Supplementary Figure S4.

(**A**) Temperature-dependent absorption spectra of **2**/**6** mixture (Q band) observed during the cooling process: [**2**]+[**6**] = 50 μM in MCH; [**6**]/{[**2**]+[**6**]} = 90%. (**B**) Change in the degree of aggregation of **2**/**6** J aggregate as a function of temperature obtained by fitting the apparent absorption coefficients at λ = 561 nm to the isodesmic model (Supplementary Equation 1)[3] in which $\alpha_{\mathbf{Mono}}$ is the degree of **2**/**6**$_{\mathbf{Mono}}$ (monomeric **2**/**6** mixture), $\varepsilon_{\mathbf{Mono}}$ and ε_J are the molar absorption coefficients of **2**/**6**$_{\mathbf{Mono}}$ and **2**/**6** J aggregate, respectively, and ε (T) is the apparent absorption coefficient at the given temperature. The fitting to the isodesmic model yielded $\Delta H°$ and T_m as summarized in Supplementary Table 1 (correlation coefficient of 0.999). (**C**) Degree of polymerization number of **2**/**6** mixture as a function of the temperature obtained by the isodesmic model (Supplementary Equation 2)[3]. (**D**) Plot of the natural logarithm of K_I as a function of the reciprocal temperature (T^{-1}): van't Hoff plot showing a linear relationship (correlation coefficient of 0.997). The standard enthalpy change ($\Delta H°$) and standard entropy change ($\Delta S°$) for the formation of **2**/**6** J aggregate were determined using Supplementary Equation 3 in which R is the ideal gas constant.

$$\alpha_J = 1 - \alpha_{Mono} = 1 - \frac{\varepsilon(T) - \varepsilon_J}{\varepsilon_{Mono} - \varepsilon_J} = \frac{1}{1 + exp\left[-0.908\Delta H^{\circ}\ \frac{T - T_m}{RT_m^2}\right]} \qquad (1)$$

$$DP_N = \frac{1}{\sqrt{1 - \alpha(T)}} = \frac{1}{2} + \frac{1}{2}\sqrt{4K_I(T)c_T + 1} \qquad (2)$$

$$\ln K_I = \frac{-\Delta H^{\circ}}{RT} + \frac{\Delta S^{\circ}}{R} \qquad (3)$$

Summary of thermodynamic analyses of **2**/**6** J aggregate

Supplementary Table S1. Thermodynamic parameters.

	isodesmic model[3]		van't Hoff plot		
[**6**]/{[**2**]+[**6**]} (%)	ΔH° (kJ mol^{-1})	T_m (K)	ΔH° (kJ mol^{-1})	ΔS° (J mol^{-1} K^{-1})	ΔG° at 308 K (kJ mol^{-1})
**100**	−81	322	−82	−176	−27.5
**90**	−83	323	−83	−183	−27.6
**80**	−83	322	−84	−185	−27.6
**70**	−83	323	−84	−184	−27.7

Time-dependent evolution of metastable two-component **2**/**6** J aggregate.

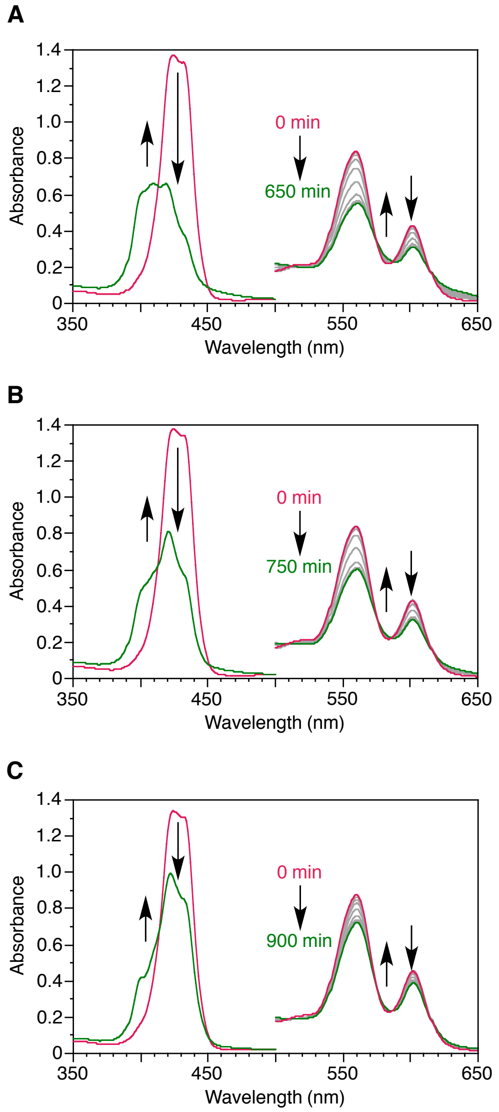

Supplementary Figure S5.

(**A**–**C**) Time-dependent absorption spectral changes of two-component **2**/**6** J aggregate : [**2**]+[**6**] = 50 μM; [**6**]/{[**2**]+[**6**]} = (**A**) 90%, (**B**) 80%, and (**C**) 70%.

Thermodynamic analysis of **5**/**6** J aggregate

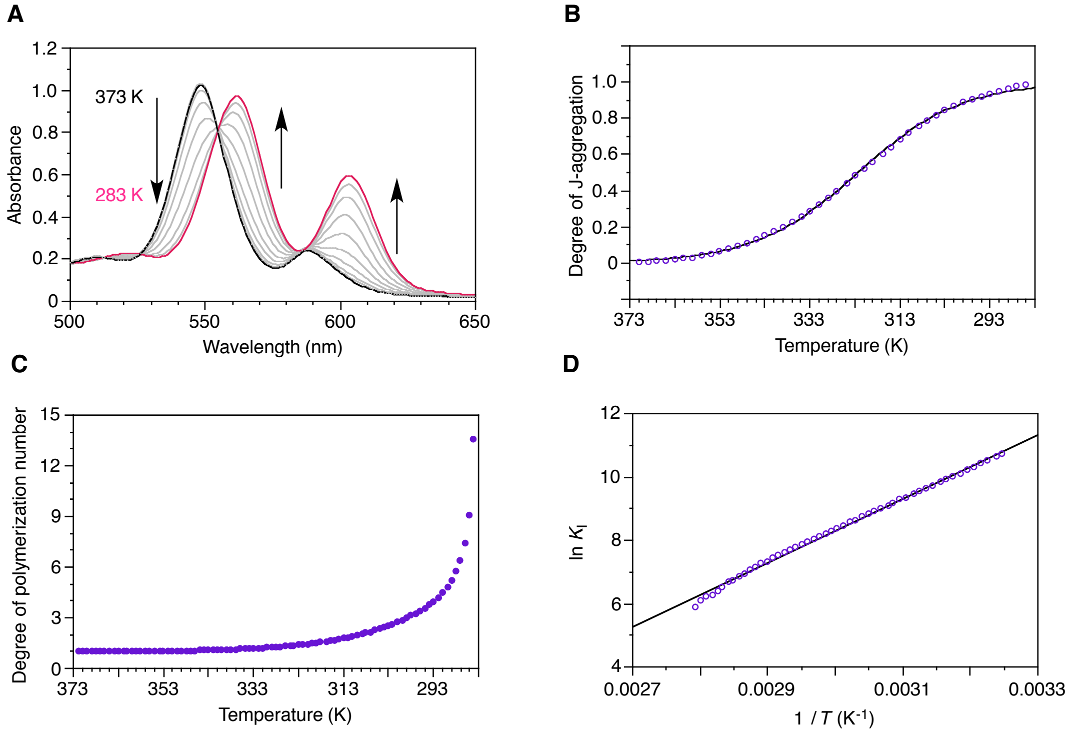

Supplementary Figure S6.

(**A**) Temperature-dependent absorption spectra of **5**/**6** mixture (Q band) observed during the cooling process: [**5**]+[**6**] = 50 μM in MCH; [**6**]/{[**5**]+[**6**]} = 50%. (**B**) Change in the degree of aggregation of **5**/**6** J aggregate as a function of temperature obtained by fitting the apparent absorption coefficients at λ = 561 nm to the isodesmic model (Supplementary Equation 1)[3] in which $\alpha_{\mathbf{Mono}}$ is the degree of **5**/**6**$_{\mathbf{Mono}}$ (monomeric **5**/**6** mixture), $\varepsilon_{\mathbf{Mono}}$ and ε_J are the molar absorption coefficients of **5**/**6**$_{\mathbf{Mono}}$ and **5**/**6** J aggregate, respectively, and ε (T) is the apparent absorption coefficient at the given temperature. The fitting to the isodesmic model yielded ΔH and T_m as summarized in Supplementary Table 2 (correlation coefficient of 0.999). (**C**) Degree of polymerization number of **5**/**6** mixture as a function of the temperature obtained by the isodesmic model (Supplementary Equation 2)[3].(**D**) Plot of the natural logarithm of K_I as a function of the reciprocal temperature (T^{-1}): van 't Hoff plot showing a linear relationship (correlation coefficient of 0.997). The standard enthalpy change ($\Delta H°$) and standard entropy change ($\Delta S°$) for the formation of **5**/**6** J aggregate were determined using Supplementary Equation 3 in which R is the ideal gas constant.

Summary of thermodynamic analyses of **5**/**6** J aggregate

Supplementary Table S2. Thermodynamic parameters.

	isodesmic model[3]		van't Hoff plot		
[**6**]/{[**5**]+[**6**]} (%)	*ΔH*° (kJ mol^{-1})	T_m (K)	*ΔH*° (kJ mol^{-1})	*ΔS*° (J mol^{-1} K^{-1})	*ΔG*° at 308 K (kJ mol^{-1})
**100**	−81	322	−82	−176	−27.5
**90**	−82	322	−83	−178	−27.6
**80**	−82	322	−83	−181	−27.5
**70**	−82	322	−82	−176	−27.5
**60**	−82	322	−83	−181	−27.6
**50**	−83	322	−84	−184	−27.6
**40**	−81	323	−82	−176	−27.6
**30**	−82	322	−83	−179	−27.6
**20**	−81	323	−80	−172	−27.6
**10**	−81	323	−82	−176	−27.6

Time-dependent evolution of metastable two-component **5**/**6** J aggregate.

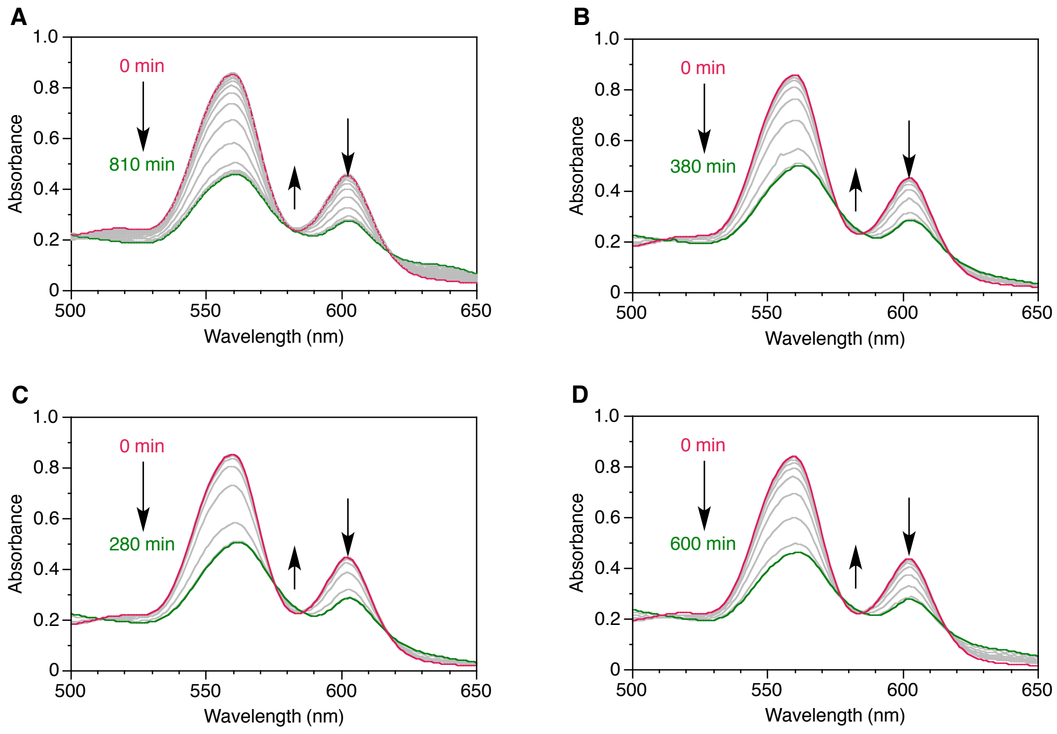

Supplementary Figure S7.

(**A**–**D**) Time-dependent absorption spectra changes of two-component **5**/**6** J aggregate : [**5**]+[**6**] = 50 μM; [**6**]/{[**5**]+[**6**]} = (**A**) 0%, (**B**) 10%, (**C**) 50%, and (**D**) 90%.

The stability of **5**/**6** H aggregate

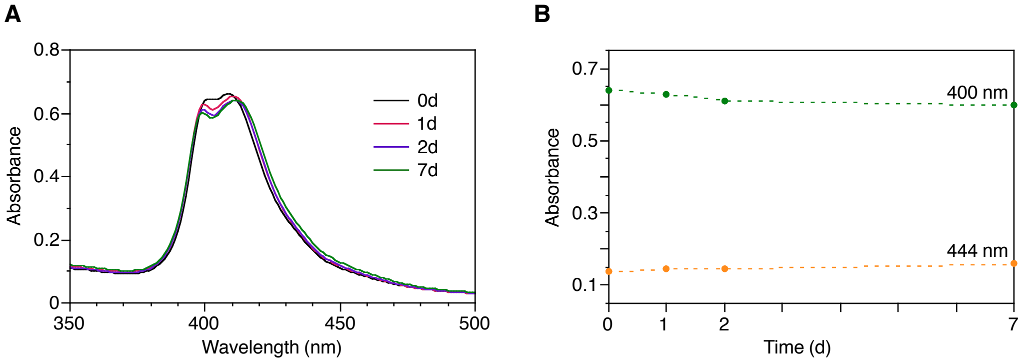

Supplementary Figure S8.

(A) Time-dependent absorption spectra changes of **5**/**6** H aggregate: [**5**]+[**6**] = 50 μM; [**6**]/{[**5**]+[**6**]} = 50%. (**B**) Time profile of changes in the absorbance at 400 nm and 444 nm. Absorbance at 400 nm and 444 nm are characteristic of the H aggregate and the short-slipping J aggregate, respectively.

The pseudo-self-sorted system of H aggregate and short-slipping J aggregate

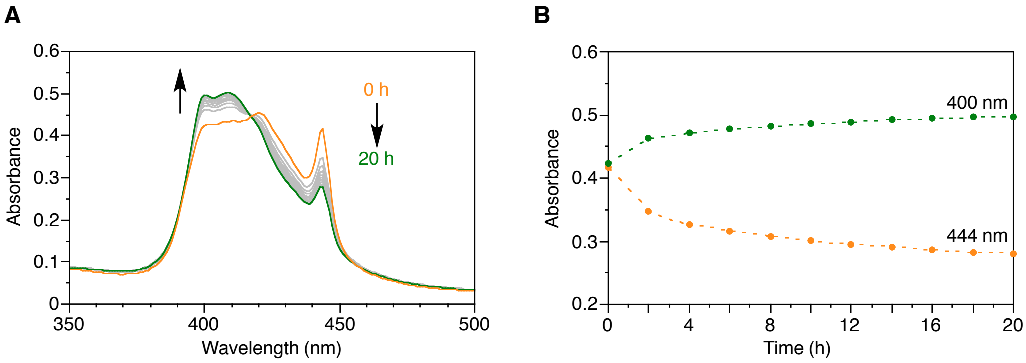

Supplementary Figure S9.

(**A**) Time-dependent absorption spectra changes of mixture of **5** H aggregate and **6** short-slipping J aggregate: [**5**]+[**6**] = 50 μM; [**6**]/{[**5**]+[**6**]} = 50%. (**B**) Time profile of changes in the absorbance at 400 nm and 444 nm. Absorbance at 400 nm and 444 nm are characteristic of the H aggregate and the short-slipping J aggregate, respectively.

Time-dependent evolution of **5**/**6** J aggregate.

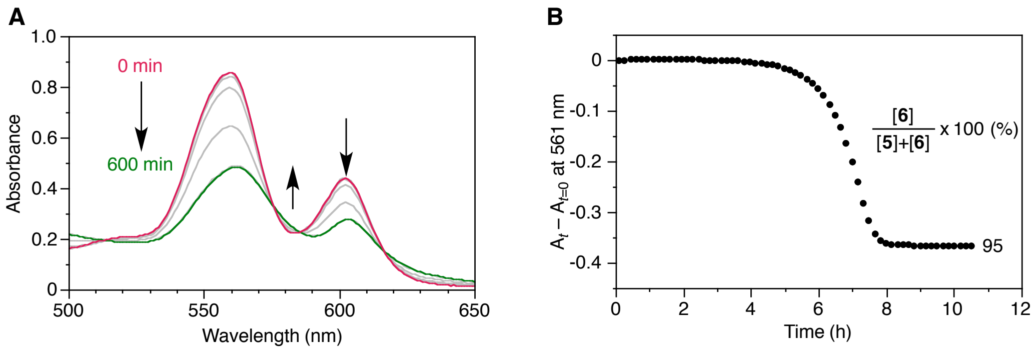

Supplementary Figure S10.

(**A**) Time-dependent absorption spectra changes of two-component **5**/**6** J aggregate : [**5**]+[**6**] = 50 μM, [**6**]/{[**5**]+[**6**]} = 95%. (**B**) Time profile of change in the absorbance at 561 nm, a wavelength that is characteristic of the H aggregate: [**5**]+[**6**] = 50 μM, [**6**]/{[**5**]+[**6**]} = 95%.

3. Supplementary References

1. Ogi, S., Fukui, T., Jue, M. L., Takeuchi, M. & Sugiyasu, K. Kinetic control over pathway complexity in supramolecular polymerization through modulating the energy landscape by rational molecular design. *Angew. Chem. Int. Ed.* **53**, 14363-14367 (2014).
2. Fukui, T. *et al.*, Control over metastable supramolecular assembly in one and two dimensions. *Nat. Chem.* (doi:10.1038/nchem2684).
3. Smulders, M. M. J. *et al.* How to distinguish isodesmic from cooperative supramolecular polymerisation. *Chem. Eur. J.* **16**, 362–367 (2010).
